# [10]-gingerol induces apoptosis and inhibits metastatic dissemination of triple negative breast cancer *in vivo*

**DOI:** 10.18632/oncotarget.20139

**Published:** 2017-08-10

**Authors:** Ana Carolina B.M. Martin, Angelina M. Fuzer, Amanda B. Becceneri, James Almada da Silva, Rebeka Tomasin, Delphine Denoyer, Soo-Hyun Kim, Katherine A. McIntyre, Helen B. Pearson, Belinda Yeo, Aadya Nagpal, Xiawei Ling, Heloisa S. Selistre-de-Araújo, Paulo Cézar Vieira, Marcia R. Cominetti, Normand Pouliot

**Affiliations:** ^1^ Department of Gerontology, Federal University of São Carlos, São Carlos, SP, Brazil; ^2^ Department of Chemistry, Federal University of São Carlos, São Carlos, SP, Brazil; ^3^ Metals in Medicine Laboratory, Centre for Cellular and Molecular Biology (CCMB), Melbourne Burwood Campus, Deakin University, VIC, Australia; ^4^ Department of Pathology and University of Melbourne, VIC, Australia; ^5^ Sir Peter MacCallum Department of Oncology, University of Melbourne, VIC, Australia; ^6^ European Cancer Stem Cell Research Institute, Cardiff University, Cathays, Cardiff, UK; ^7^ Matrix Microenvironment and Metastasis Laboratory, Olivia Newton-John Cancer Research Institute, School of Cancer Medicine, La Trobe University, Heidelberg, Australia; ^8^ Department of Physiological Sciences, Federal University of São Carlos, São Carlos, SP, Brazil

**Keywords:** gingerol, breast cancer, cell cycle, apoptosis, animal models

## Abstract

There is increasing interest in the use of non-toxic natural products for the treatment of various pathologies, including cancer. In particular, biologically active constituents of the ginger oleoresin (*Zingiber officinale* Roscoe) have been shown to mediate anti-tumour activity and to contribute to the anti-inflammatory, antioxidant, antimicrobial, and antiemetic properties of ginger. Here we report on the inhibitory properties of [10]-gingerol against metastatic triple negative breast cancer (TNBC) *in vitro* and *in vivo*. We show that [10]-gingerol concentration-dependently induces apoptotic death in mouse and human TNBC cell lines *in vitro*. In addition, [10]-gingerol is well tolerated *in vivo*, induces a marked increase in caspase-3 activation and inhibits orthotopic tumour growth in a syngeneic mouse model of spontaneous breast cancer metastasis. Importantly, using both spontaneous and experimental metastasis assays, we show for the first time that [10]-gingerol significantly inhibits metastasis to multiple organs including lung, bone and brain. Remarkably, inhibition of brain metastasis was observed even when treatment was initiated after surgical removal of the primary tumour. Taken together, these results indicate that [10]-gingerol may be a safe and useful complementary therapy for the treatment of metastatic breast cancer and warrant further investigation of its efficacy, either alone or in combination with standard systemic therapies, in pre-clinical models of metastatic breast cancer and in patients.

## INTRODUCTION

Breast cancer is the third most diagnosed cancer worldwide and the second highest cause of death among women in developing countries [1]. TNBC that accounts for 10–20% of cases is characterised by rapid progression to metastasis [2–4]. Patients with TNBC do not respond to endocrine or HER2-targeted therapies due to the lack of oestrogen, progesterone or HER2 receptor expression [5]. Whilst initially responsive to chemotherapy, relapse is common, with the emergence of chemo-resistant metastases inevitably resulting in death [6]. Up to 30% of advanced TNBC patients develop incurable brain metastases for which chemotherapy provides little benefit, in part due to poor ability of most chemotherapeutic agents to cross the blood-brain barrier [7, 8]. Thus, the limited long-term efficacy of chemotherapy in advanced TNBC patients and its normal tissue toxicity remain primary concerns [9, 10], motivating the search for natural products with fewer side-effects as an alternative or complementary chemotherapy for metastatic breast cancer and other malignancies [11–13].

Gingerols, the major pungent constituents in the ginger (*Zingiber officinale* Roscoe) oleoresin from fresh rhizome, comprise a series of homologues differentiated by the length of their alkyl chains, with [6]-gingerol being the most abundant [14]. Increasing evidence indicates that gingerols, especially [6]-gingerol, mediate anti-tumour and anti-metastatic activity *in vitro* and *in vivo* against a variety of tumour types, including breast cancer [15]. Fewer studies have investigated the anti-tumour properties of [10]-gingerol and relatively little is known about its mechanism of action. In colon cancer cells, [10]-gingerol induces cell cycle arrest, elevation of intracellular Ca^2+^ and caspase-dependent apoptosis [16, 17]. More recently, Joo and colleagues [18] reported that inhibition of MDA-MB-231 breast cancer cell proliferation and invasion by [10]-gingerol is associated with inactivation of AKT and p38MAPK. However, to our knowledge, its anti-tumour activity *in vivo* has not been investigated, most likely due to its lower abundance and difficulty of purifying sufficient biologically active [10]-gingerol.

Previously, we reported a new methodology for efficient isolation and purification of [10]-gingerol by reverse-phase HPLC [19]. Our study showed that [10]-gingerol is a more potent inhibitor than [6]- or [8]-gingerol, with selectivity towards breast cancer cells compared to normal fibroblasts *in vitro*. In addition, more recently we have demonstrated that [10]-gingerol is able to revert the malignant phenotype of breast cancer cells in three-dimensional culture [20]. Interestingly, [10]-gingerol also mediates potent anti-neuroinflammatory activity [21], an important manifestation likely to contribute to the development of brain metastases [22]. Therefore, [10]-gingerol could potentially benefit breast cancer patients with brain metastasis through its direct anti-tumour and/or neuro-protective properties. Here, we show that [10]-gingerol induces apoptosis in aggressive mouse and human TNBC cells. Importantly, using a clinically relevant syngeneic model of breast cancer brain metastasis, we demonstrate for the first time that [10]-gingerol inhibits tumour growth and spontaneous metastasis to multiple organs, including lung, bone and brain.

## RESULTS

### [10]-gingerol induces apoptosis of metastatic TNBC cells

The activity of [10]-gingerol against brain metastatic cells that are typically more resistant to therapy has not been reported. We initially compared the effect of [10]-gingerol on mouse (4T1Br4) and human (MDA-MB-231BrM) brain metastatic tumour variants versus isogenic lines that are highly metastatic to lung and/or bone but not to brain (4T1BM2 and MDA-MB-231).

**Figure 1:**
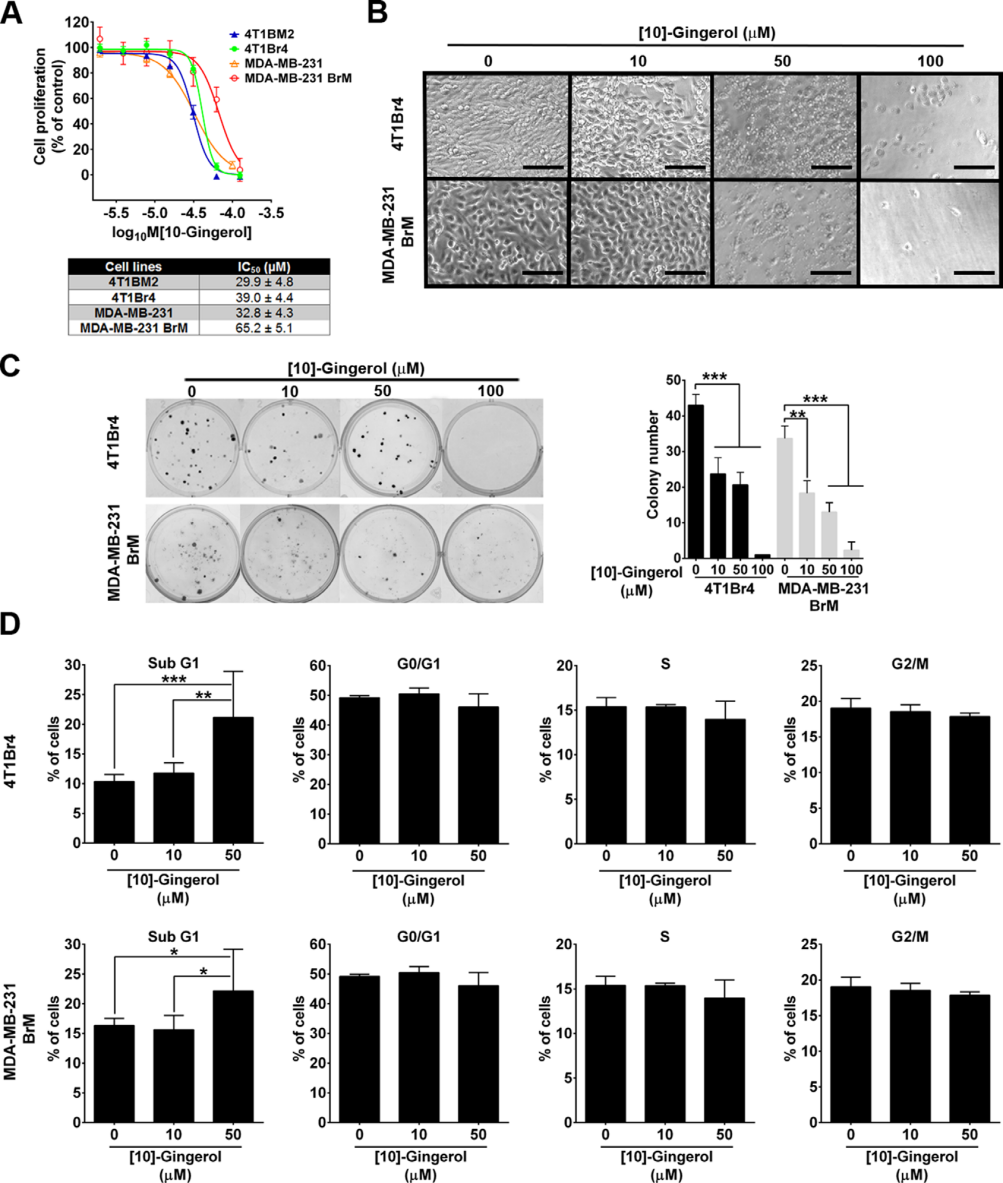
[10]-gingerol induces concentration-dependent cell death in mouse and human metastatic TNBC cells *in vitro* (**A**) Differential sensitivity of mouse and human metastatic TNBC cells to [10]-gingerol. Cells were treated with increasing concentrations of [10]-gingerol and proliferation measured after 3 days using the SRB colorimetric assay. Data show mean ± SD of 6 replicates/dose from a representative experiment (*n* = 3). IC_50_ values for each cell line are indicated in the bottom panel. (**B**) [10]-gingerol induces morphological changes and cell detachment *in vitro*. Confluent monolayers of 4T1Br4 and MDA-MB-231BrM were treated with the indicated concentrations of [10]-gingerol and changes in cell morphology and adherence were visualised after 24h on an inverted microscope. Note the significant loss of membrane integrity (50 μM) and reduced number adherent cells with 100 μM [10]-gingerol treatment. Scale bar, 50 μm. (**C**) Effects of [10]-gingerol on colony formation at low cell density. 4T1Br4 (100 cells/well) and MDA-MB-231BrM cells (300 cells/well) were seeded in 6-well plates, allowed to adhere overnight at 37°C and treated with indicated concentrations of [10]-gingerol or vehicle (DMSO) alone for 24 h. Colonies (> 50 cells) formed after 8 days were stained with a solution of crystal violet, photographed and counted. Data show representative wells at each [10]-gingerol concentration (left panel). Assays were repeated three times in triplicates and data show mean ± SD of triplicates from a representative experiment (right panel). **p < 0.01, ***p < 0.001, 1-way ANOVA, Bonferroni post-test. (**D**) Effect of [10]-gingerol on cell cycle. Adherent 4T1Br4 (top panels) and MDA-MB-231Br cells (bottom panels) were treated for 24h with indicated concentrations of [10]-gingerol, processed for cell cycle analysis and DNA content analysed by flow cytometry as described in Materials and methods. Data show mean % of cells in each cycle phase ± SD from 3 experiments and statistical difference between treatment groups for each phase of the cell cycle analysed by 2-way ANOVA and Tukey's multiple comparison test. *p < 0.05, **p < 0.01, ***p < 0.001.

[10]-gingerol inhibited the proliferation of all cell lines tested (Figure 1A). However, brain-metastatic variants were less sensitive than non-brain metastatic lines, as evidenced by their higher IC_50_ values (Figure 1A). Treatment with [10]-gingerol for 24 h caused significant cell death and detachment of confluent 4T1Br4 or MDA-MB-231BrM cultures at high concentration (100 μM), whereas [10]-gingerol had a partial effect on cell viability and attachment at 50 μM and only negligible effect at low concentration (10 μM) (Figure 1B). Similarly, [10]-gingerol concentration-dependently inhibited colony formation at low cell density, with partial inhibition at 10 μM and 50 μM and almost complete inhibition at 100 μM (Figure 1C). Consistent with a cytotoxic activity rather than an anti-proliferative activity of [10]-gingerol, cell cycle analyses in either cell line revealed only marginal changes in the proportion of cells in G0/G1, S phase or G2/M phase but accumulation of cells in sub-G1 after 24 h treatment with 50 μM [10]-gingerol (Figure 1D). Attempts to measure cell cycle phases after 24h treatment with 100 μM [10]-gingerol gave inconsistent results due to the extensive cell death and loss of DNA during processing of both cell lines (data not shown).

**Figure 2:**
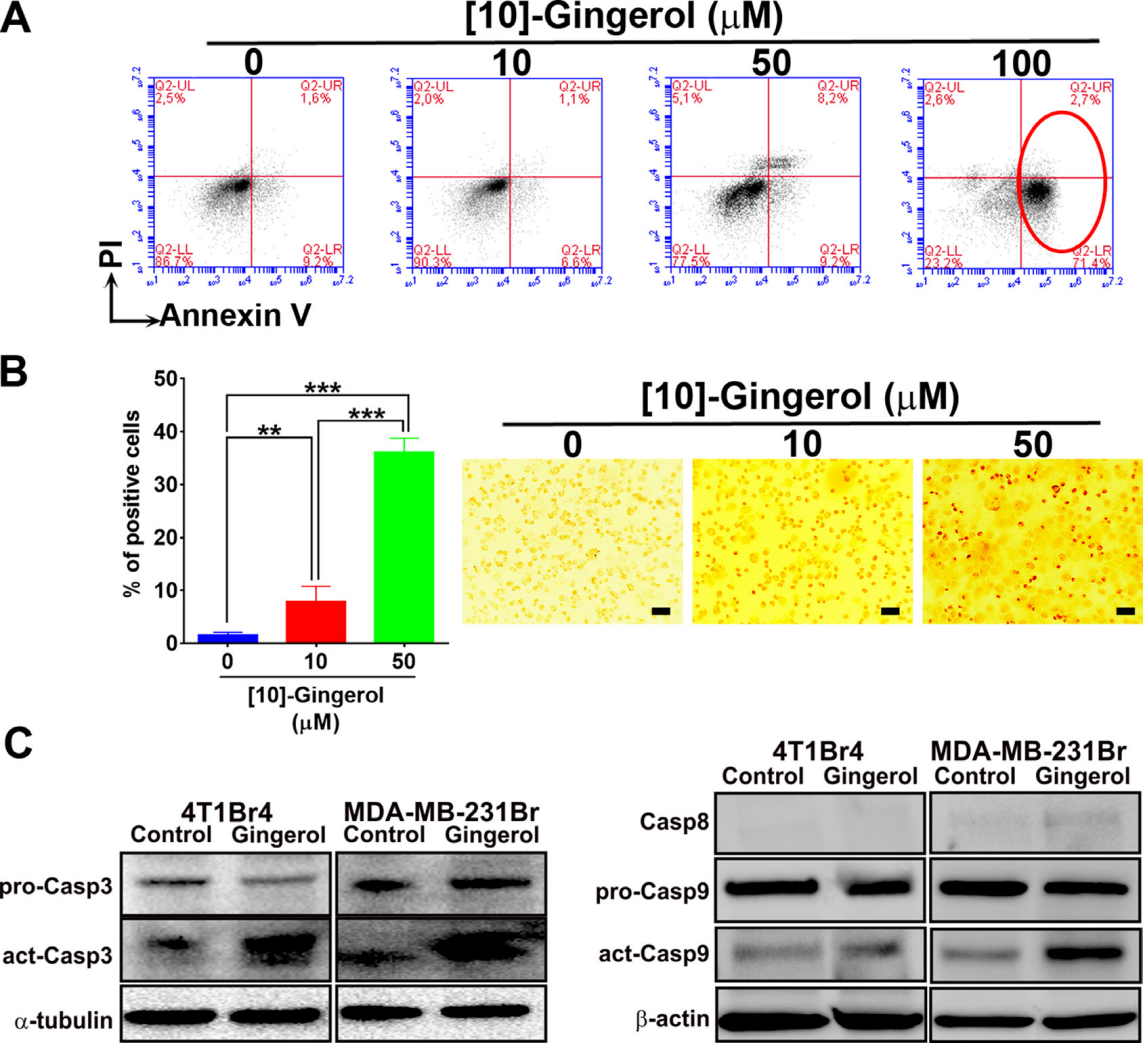
[10]-gingerol induces concentration-dependent apoptosis in metastatic TNBC cells *in vitro* (**A**) Flow cytometric analysis of Annexin-V in [10]-gingerol-treated 4T1Br4 cells. 4T1Br4 cells were treated with 0, 10, 50 and 100 μM [10]-gingerol for 8 h, fixed and stained with PE-Annexin-V and 7-AAD as described in Supplementary methods. Annexin-V positive cells are indicated with a red circle and the percentage of early and late apoptotic cells are indicated in the lower and upper right quadrants respectively. (**B**) TUNEL positivity was determined after 18h treatment with 10 or 50 μM of [10]-gingerol as indicated and as described in Supplementary methods. Representative images are shown on the left. Scale bar = 100 μm. The number of positive cells were counted and the data expressed as mean % of positive cells ± SD from six 10× images/condition (right panel). Statistical significance was determined using a 1-way ANOVA, Bonferroni post-test, **p < 0.01, ***p < 0.001. (**C**) Caspase expression following [10]-gingerol treatment *in vitro*. Expression of pro- and activated caspases in 4T1Br4 and MDA-MB-231BrM cells was analysed by western blotting after 6 h exposure to [10]-gingerol (50 μM) as described in Materials and Methods. α-tubulin or β-actin was used as loading control as indicated.

To confirm that [10]-gingerol induces apoptosis, 4T1Br4 cells were treated with 10 μM, 50 μM or 100 μM [10]-gingerol for 8h, stained for PE-Annexin-V and analysed by flow cytometry. [10]-gingerol induced a concentration-dependent increase in early and late apoptotic cells (Figure 2A). This was confirmed by TUNEL staining showing approximately 40% apoptosis after treatment with [10]-gingerol (50 μM) for 18 h (Figure 2B). Similar observations were made in MDA-MB-231BrM cells (Supplementary Figure 1). Early induction of caspase activation was analysed by western blotting on cell lysates from control and 50 μM [10]-gingerol-treated cells for 6 h (Figure 2C). We found that [10]-gingerol induced potent activation of caspase-3 and caspase-9 in both cell lines under these conditions. In contrast, only low levels of caspase-8 were detected and did not change significantly between control and [10]-gingerol-treated cells.

### [10]-gingerol inhibits 4T1Br4 orthotopic tumour growth and spontaneous metastasis

**Figure 3:**
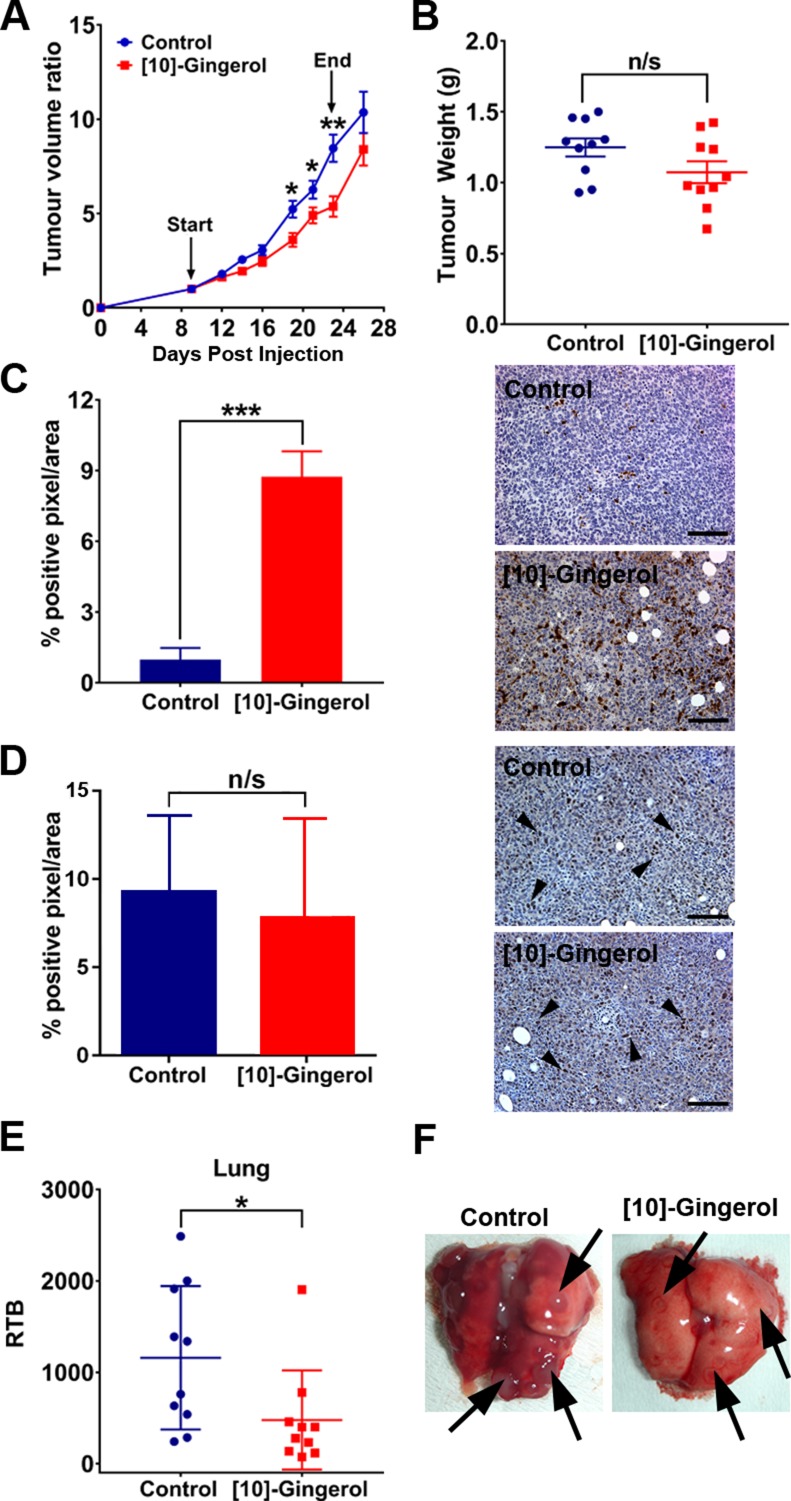
[10]-gingerol delays orthotopic tumour growth and inhibits spontaneous metastasis (**A**) Tumour growth rate. 4T1Br4 cells (1 × 10^5^) were inoculated into the mammary fat pad and vehicle (saline) or [10]-gingerol (5 mg/Kg) was administered daily intraperitoneally, from day 9 to day 23. Primary tumour growth was measured thrice weekly using electronic callipers. Start and end of treatment are indicated with arrows. Difference in growth rate between groups (*n* = 10 mice/group) was analysed by 2-way ANOVA, Bonferroni post-test (*p < 0.05; **p < 0.01). (**B**) Tumour weight at endpoint (day 26). Data show one point for each mouse (*n*= 10/group) and mean burdens (horizontal bar) ± SD. n/s = not significant, Mann Whitney test (*p* = 0.109). (**C**) Detection of active caspase-3 and (D) Ki67 in primary tumours. IHC staining of primary tumour sections was carried out as described in Supplementary methods. A total of 27 images/experimental group (3 images/section x 3 section/tumour 150 μm apart × 3 tumours/group) was analysed. Arrowheads in (**D**) indicate positive Ki67 nuclear reactivity. Data are expressed as mean % of positive pixels/field of view ± SD and the statistical difference between groups was analysed using Mann Whitney test; ***p < 0.001. Representative images of control and [10]-gingerol-treated mice are shown on the right. Scale bar = 100 μm. (**E**) Spontaneous lung metastasis. Relative tumour burden (RTB) in lung was quantitated by genomic qPCR detection of mCherry gene relative to vimentin as described in Materials and methods. Data show one point for each mouse and mean burdens (horizontal bar) ± SD. *p < 0.001, Mann Whitney test. (**F**) Representative images of lungs from control (saline) and [10]-gingerol-treated mice. Arrows indicate metastatic nodules.

We validated the anti-tumour effects of [10]-gingerol *in vivo* using metastatic 4T1Br4 tumours. [10]-gingerol (5 mg/Kg) was administered daily from day-9 to day-23 and the mice sacrificed on day-26. At this dose, [10]-gingerol was well tolerated, with no significant bodyweight loss (Supplementary Figure 2A). Gross histological examination of livers and kidneys and H&E staining revealed no evident toxicity (Supplementary Figure 2B). Importantly, tumour growth was partially inhibited, with inhibition being most evident between days 19–23 (Figure 3A). Treatment was stopped on day 23 when control mice began showing early signs of ill-health due to metastatic disease and the experiment was terminated when control mice showed clear signs of high metastatic burden. As expected, tumour growth resumed upon cessation of [10]-gingerol treatment and tumour weight was not significantly different at endpoint (day 26) (Figure 3B). However, IHC staining of cleaved (active) caspase-3 in primary tumours showed a dramatic increase in the number of apoptotic cells in [10]-gingerol-treated mice (Figure 3C). Moreover, immunostaining of the proliferation marker Ki67 in primary tumours did not show a significant difference between control and [10]-gingerol-treated mice (Figure 3D). Metastatic burden in lung was significantly decreased by [10]-gingerol (Figure 3E, 3F), with a trend towards reduced bone metastasis as well (data not shown).

**Figure 4:**
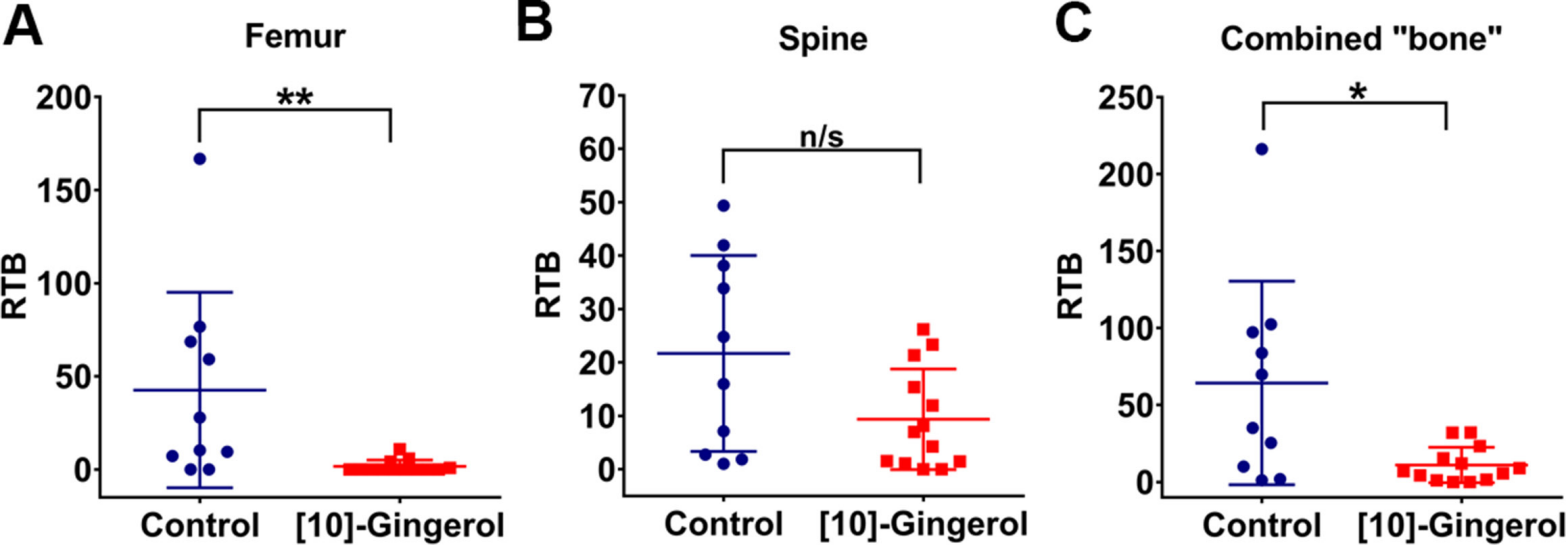
[10]-gingerol inhibits 4T1 experimental metastasis to bone 4T1Br4 cells (5 × 10^4^) were inoculated into the left ventricle of the heart and the mice treated with daily IP injections of vehicle (saline) or [10]-gingerol (10 mg/Kg) as described in Materials and methods. Mice were harvested on day 12 and relative tumour burden (RTB) in (**A**) femur, (**B**) spine and (**C**) bone (combined femur and spine) determined by genomic qPCR detection of mCherry gene relative to vimentin as described in Supplementary methods. Data show one point per mouse (control, *n* = 10; [10]-gingerol, *n* = 13) and mean burdens (horizontal bar) ± SD. *p < 0.05; **p< 0.01; ns, not significant (spine, *p* = 0.086, Mann Whitney test).

[10]-gingerol used at 5 mg/kg partially reduced bone metastasis but was not sufficient to achieve statistical significance. We reasoned that a higher dose may be required to efficiently target metastatic disease. Thus, for subsequent experiments, [10]-gingerol was used at 10 mg/kg. Bone metastasis was further analysed in an experimental metastasis assay in which the formation of a primary tumour is bypassed by direct injection of 4T1Br4 cells into the left cardiac ventricle (Figure 4). [10]-gingerol (10 mg/Kg) was administered one day after tumour cell inoculation and continued daily until completion on day 12. Under these conditions, femoral metastases were significantly inhibited (Figure 4A). While spine analysis showed a trend toward decreased metastasis (Figure 4B), combined metastatic burden scores for femurs + spine from same mice were significantly lower in [10]-gingerol-treated animals (Figure 4C).

**Figure 5:**
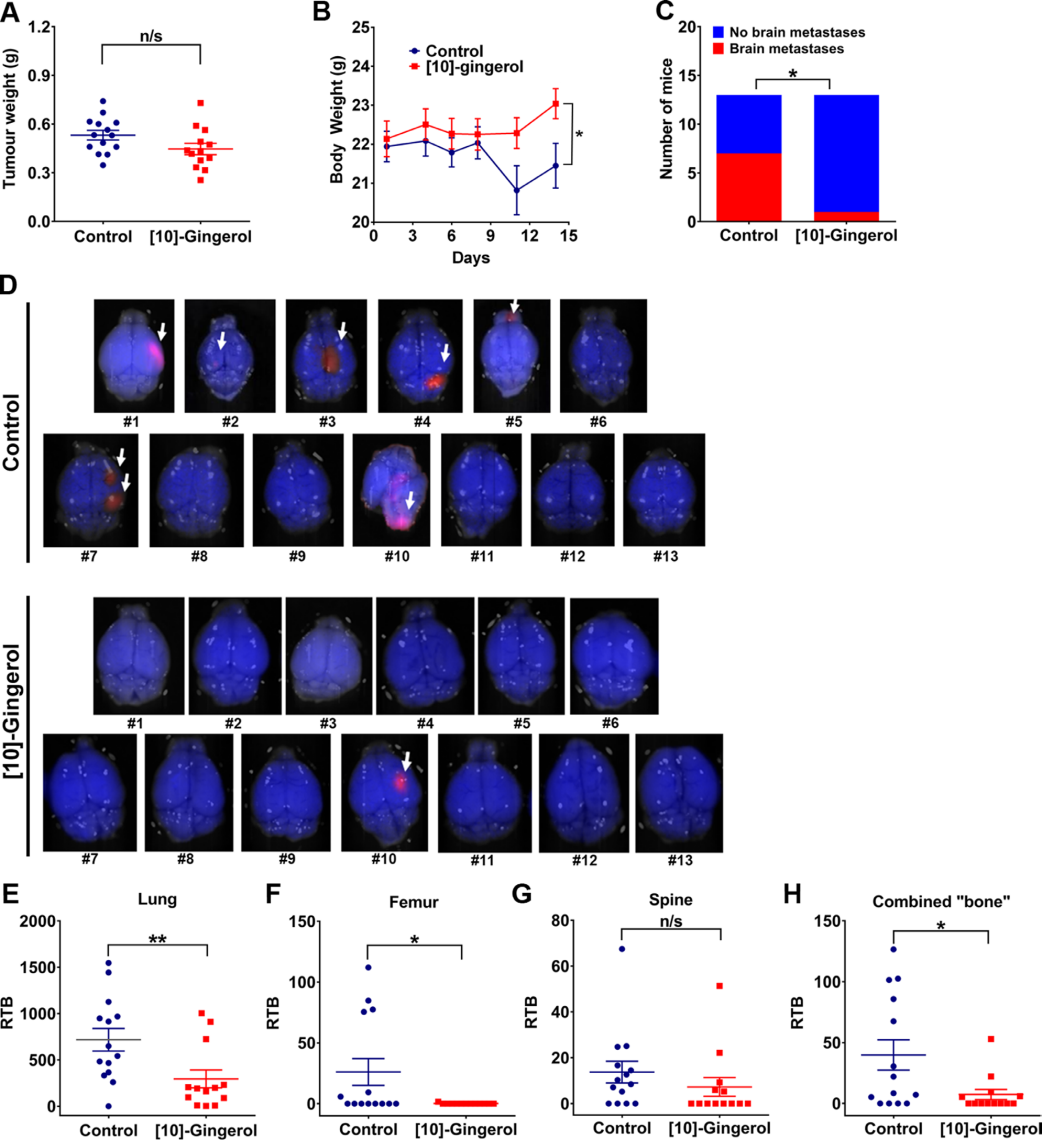
[10]-gingerol inhibits 4T1 spontaneous metastasis to brain 4T1Br4 cells (1 × 10^5^) were inoculated into the mammary fat pad and tumours surgically removed when they reached ∼0.5 cm^3^. Mice were treated for 14 days with daily IP injections of vehicle (saline) or [10]-gingerol (10 mg/Kg) starting one day after tumour resection and the mice sacrificed on day 14 for quantitation of metastatic burden. (**A**) Primary tumour weight at resection. Each point represents one mouse (Control; *n* = 14, [10]-gingerol; *n* = 13). Data were analysed for statistical significance using Mann Whitney test. n/s = not significant (*p* = 0.307). (**B**) Body weight measurements post-tumour resection. *p = 0.041, Mann Whitney test. (**C**) Incidence of mice with brain metastases. Brains were removed and analysed by fluorescence imaging for the presence of mCherry^+ve^ nodules and statistical difference between groups analysed with Fisher's exact test. *p < 0.05. (**D**) Fluorescence images of brains from each mouse from control (top) and [10]-gingerol-treated (bottom) groups. Note that control brain #14 in (C) and (D) could not be analysed due to overnight death and cannibalism. Metastatic nodules are indicated by an arrow. Metastatic burden in (**E**) lung, (**F**) femur, (**G**) spine and (**H**) combined bones (femur + spine from same mice) determined by genomic qPCR detection of mCherry gene relative to vimentin as described in Supplementary methods. Data are expressed as relative tumour burden (RTB) and show one point per mouse (control, *n* = 14; [10]-gingerol, *n* = 13) and mean burdens (horizontal bar) ± SD. *p < 0.05; **p < 0.01; ns, not significant (spine, *p* = 0.086, Mann Whitney test).

To further evaluate the clinical relevance of our findings and to test the impact of [10]-gingerol (10 mg/kg) on the development of late brain metastases, we completed an experiment where treatment commenced one day after surgical removal of the primary tumour at ∼0.5 cm^3^ (∼0.5 g) and continued for 14-days (Figure 5A). Interestingly, body weight measurement over the subsequent 14 days of treatment showed that control mice lost weight, indicative of cachexia typically observed in mice with a high tumour burden and commonly observed in advanced patients. In contrast, mice from the [10]-gingerol group gained some weight (Figure 5B). Daily monitoring of animals also indicated a healthier general appearance in the [10]-gingerol group, as evidenced by the level of activity and coat appearance compared to controls. Consistent with these observations, mCherry fluorescence imaging of brains at endpoint indicated a significantly lower incidence of mice with brain lesions in the [10]-gingerol-treated group (1/13) compared to controls (7/13) (Figure 5C, 5D). Moreover, [10]-gingerol reduced spontaneous lung and bone metastatic burden (Figure 5E–5H).

Histological examination of brain lesions from either control mice or mice treated with [10]-gingerol (10 mg/kg) by H&E and IHC staining of GFAP, revealed highly vascularised lesions and recruitment of GFAP^+ve^ activated astrocytes in the periphery of metastatic lesions, indicative of reactive glia (Supplementary Figure 3A). Surprisingly, we did not detect activated/cleaved caspase-3 in brain lesions from either control or [10]-gingerol-treated mice indicating that once established, brain metastases may become refractory to [10]-gingerol inhibition (Supplementary Figure 3B). Collectively, the results indicate that [10]-gingerol promotes apoptotic death, inhibits TNBC growth and metastasis to multiple organs *in vivo*.

## DISCUSSION

We investigated the anti-tumour/metastatic properties of [10]-gingerol against brain metastatic TNBC *in vitro* and *in vivo*. Our data demonstrate for the first time that [10]-gingerol inhibits TNBC orthotopic growth and spontaneous metastasis to multiple organs. Although [10]-gingerol only had a modest effect of primary tumour volume *in vivo*, histological examination showed more extensive cell death within mammary tumours of [10]-gingerol-treated mice. Further, we found that [10]-gingerol promotes cell death primarily via induction of caspase-dependent apoptosis, evidenced by a significant increase in caspase-3 activation throughout primary tumours, cleavage of caspase-3 and −9 *in vitro*, or accumulation of cells in sub-G1, TUNEL positivity and concentration-dependent increase in annexin-V staining *in vitro*. These observations are consistent with those made in cervical cancer cells [23] and colon cancer cells [17] in which [10]-gingerol predominantly induced time-dependent accumulation of cells in sub-G1 at similar concentrations (30–50 μM) to the IC_50_ values we have observed in TNBC cell lines. Moreover, in agreement with our results, Ryu and Chung [17] found that induction of cell death was associated with cleavage of caspase-3 and −9, indicating that [10]-gingerol induces apoptosis via the intrinsic pathway.

Interestingly, recent studies have reported that [10]-gingerol inhibits cell growth through its effect on both, cell cycle progression and survival in breast cancer cells, although the results from these studies are conflicting. One study showed that 10 μM [10]-gingerol was sufficient to partially prevent serum-induced MDA-MB-231 cell cycling [18]. Others reported that [10]-gingerol inhibits TNBC cell growth (including 4T1 and MDA-MB-231 cells) *in vitro* at concentrations ≥ 50 μM [24]. Surprisingly, the authors reported that MDA-MB-231 cells accumulate in S-phase but this effect required treatment for 72 h with concentrations of [10]-gingerol as high as 200 μM, a concentration we found to potently and rapidly induce apoptotic death in 4T1Br4 and MDA-MB-231BrM brain-metastatic variants. Moreover, Bernard and colleagues [24] found that [10]-gingerol-induced cell death was not inhibited by the broad spectrum caspase inhibitor zVAD-fmk, suggesting an additional caspase-independent mechanism. The reasons for the discrepancies between these studies and ours are unclear but could be in part due to differences in sensitivity to [10]-gingerol between brain-metastatic variants and other TNBC lines or to the methodology and time-point used to assess the impact of [10]-gingerol on breast cancer cell proliferation, survival or other cellular responses. Since caspase activation is a relatively early event, we measured changes in caspase activation *in vitro* after 6 h of treatment with 50 μM [10]-gingerol. Under those conditions, caspases-3/9 cleavage was evident whereas assays performed with higher [10]-gingerol concentrations or longer time-points gave inconsistent results (data not shown), most likely due to significant loss of cells and more advanced stage of apoptotic death.

Joo and colleagues [18] reported that inhibition of lung-metastatic MDA-MB-231 cell proliferation and invasion by [10]-gingerol is associated with suppression of Akt, p38MAPK and epidermal growth factor receptor. Whilst we have tested the impact of [10]-gingerol on long-term proliferation and migration in brain-metastatic TNBC lines, inhibition was observed only at concentrations that induce apoptotic death (data not shown) and therefore its anti-proliferative/migratory activity could not be distinguished from its effect on apoptosis. In addition, [10]-gingerol increased caspase-3 activation in tumours *in vivo* but not proliferation, evidenced by expression of the Ki67 proliferation marker, further suggesting that [10]-gingerol inhibits TNBC growth primarily by inducing apoptosis rather than inhibiting cell cycling. Whether [10]-gingerol impacts on other cellular responses and associated signalling pathways in brain-metastatic TNBC cells remains to be determined.

In metastasis assays without primary tumour resection, [10]-gingerol concomitantly inhibited orthotopic tumour growth and spontaneous lung metastasis. Whilst we cannot exclude that inhibition of metastasis was partially due to effects on primary tumours in this assay, inhibition of lung, bone and brain metastasis observed in experimental metastasis assays or when treatment commenced after resection of same size tumours indicates that [10]-gingerol impacts directly on metastatic progression. Importantly, [10]-gingerol was well tolerated, improved the overall appearance of mice and prevented weight loss due to heavy metastatic burden in bone lung and brain seen in control mice. While induction of cell death by [10]-gingerol is not rescued by treatment with antioxidants [24], it remains possible that blocking of reactive oxygen species by [10]-gingerol may contribute in part to improved general health of mice in our study and to the beneficial effects of ginger extracts in patients as suggested by others [25].

A study in healthy human volunteers showed that encapsulated ginger extract (up to 2 g of administered orally) is well tolerated [26]. However, [10]-gingerol half-life was approximately 2h and maximum plasma concentration was low (estimated at 0.53 ± 0.4 μg/ml, ∼ 1.5 μM), especially due to its diminished amount in the extract. Similarly, Mukkavilli and colleagues [27] recently reported that [10]-gingerol is stable and suitable for oral administration in mice but that it has poor solubility in plasma and rapid clearance *in vivo*. Together these studies suggested that tumour inhibition may not be achievable *in vivo* when [10]-gingerol is administered orally. However, these studies differ from ours in many respects. In particular, we administered purified [10]-gingerol daily by intra-peritoneal injections. It is conceivable that [10]-gingerol, while cleared rapidly in plasma, may accumulate in tumours upon repeated dosage. This was not investigated in the above studies but might explain the effects observed in 4T1Br4 tumour-bearing mice.

Our results showed particularly impressive inhibition of brain metastasis by [10]-gingerol. This is of significance given the syngeneic nature of the 4T1Br4 model and the important regulatory role of immune cells in brain metastasis [28]. Surprisingly, despite significantly lower incidence of brain metastasis, lesions that developed in treated animals showed the presence of a reactive glia without appreciable levels of active caspase-3 indicating either acquisition of resistance in established lesions and/or failure to achieve effective therapeutic concentration in the brain. Despite their recognised neuro-protective properties, the permeability of [10]-gingerol and related compounds across the blood-brain barrier and whether therapeutic concentration can be achieved in the brain remains contentious [21, 29, 30]. The absence of activated caspase-3 in established 4T1Br4 brain lesions from treated mice suggests that [10]-gingerol may be more effective as a ‘preventive’ complementary therapy, targeting circulating tumour cells (CTCs) and delaying their homing to brain or by inhibiting disseminated tumour cells prior to the development of macro-metastases.

Given the high tolerability and relatively short half-life of [10]-gingerol (∼2 h) [31], we are currently investigating whether greater efficacy against established brain metastases could be achieved by increasing the dose and/or frequency of treatment. Moreover, in light of the relatively low plasma concentrations achieved in human or mouse by oral administration (due in part to limited absorption and solubility), greater efficacy of [10]-gingerol against CTCs or disseminated tumour cells could potentially be achieved by improving absorption via better oral formulations and/or by strategies to improve the bioavailability of free [10]-gingerol in plasma as recently proposed [27].

Collectively, our results demonstrated that [10]-gingerol inhibits TNBC growth and spontaneous metastasis through induction of apoptosis and warrant further evaluation of [10]-gingerol in pre-clinical models and in patients. In particular, it will be important in future experiments to determine whether [10]-gingerol treatment in a neo-adjuvant or adjuvant setting could extend survival in pre-clinical animal models, either alone or in combination with standard systemic therapies. The robust and clinically relevant 4T1Br4 brain metastasis model gives rise to a high incidence of spontaneous brain (and extra-cranial) metastases and will be a valuable tool to further evaluate the efficacy and mechanism of action of [10]-gingerol or other natural compounds against incurable brain metastases.

## MATERIALS AND METHODS

### Cell culture and reagents

Murine brain-metastatic 4T1Br4 mammary tumour cells, derived from parental 4T1 cells (obtained from Dr. F. Miller, Karmanos Cancer Institute, Detroit, MI, USA) and stably transduced with a pMSCV-mCherry retroviral vector [32] were isolated by serial *in vivo* passaging [33]. Briefly, 4T1 cells were inoculated into the 4th mammary fat pad and the mice sacrificed after 32 days. A rare brain metastasis was isolated, expanded in culture and mCherry^+ve^ cells sorted by fluorescence activated cell sorting. This procedure was repeated four times to generate the 4T1Br4 variant. 4T1Br4 cells and tumours lack expression of HER2, oestrogen and progesterone receptors and are classified as TNBC [33]. 4T1Br4 and 4T1BM2 [34] were cultured as described [34] and limited to four weeks in culture. MDA-MB-231 and its brain-metastatic variant, MDA-MB-231BrM, were from Prof Joan Massague (Memorial Sloan Kettering Cancer Center, NY, USA) and cultured in DMEM, 10% foetal bovine serum, sodium pyruvate (1 mM), glutamine (2 mM) and 1% penicillin-streptomycin. [10]-gingerol was purified as described [19]. Stock solutions were prepared in DMSO (100 mM) and diluted in saline for *in vitro* and *in vivo* assays. Controls were treated with the same concentrations of DMSO alone and did not exceed 0.1% final.

### Determination of [10]-gingerol's 50% inhibitory concentration (IC_50_)

The cells were seeded in complete medium (1 × 10^3^/200 μl/well) in 6 replicate wells of 96-well plates, allowed to adhere for 6h and the medium changed to fresh medium supplemented with serial dilution of [10]-gingerol (200 μl/well, final volume). Cell proliferation was measured after 3 days using a sulforhodamine B (SRB) colorimetric assay [35]. Briefly, adherent cells were fixed by addition of 50 μl of trichloroacetic acid (TCA) and stained with a solution of 0.4% SRB dissolved in 1% acetic acid (100 μl/well). Protein-bound dye was released by addition of 100 μl of 10 mM Tris base and the absorbance measured by spectrophotometry at 550nm. Assays were completed three times and the data presented as the mean ± SD of a representative experiment. IC_50_ calculations were performed using Hill's equation in the GraphPad Prism 6.0 software.

### Colony formation assays

MDA-MB-231BrM and 4T1Br4 cells were plated in triplicate in 6 well plates at low density (300 and 100 cells, respectively) and incubated overnight at 37°C. Adherent cells were treated with [10]-gingerol or vehicle (DMSO) for 24 h in 4 ml of serum-containing medium. Colonies (> 50 cells) formed after 8 days were fixed with methanol, stained with crystal violet and counted. Statistical difference between groups was analysed using a 1-way ANOVA, Bonferroni post-test; *p* < 0.05 was considered significant.

### Cell cycle analysis

Cells (1 × 10^6^) were seeded in 6 cm dishes and incubated for 24 h at 37°C, 5% CO_2_. The cells were treated with 0, 10 or 50 μM of [10]-gingerol and incubated for a further 24 h. Cells were detached and pelleted by centrifugation, washed with cold PBS, fixed in 70% cold ethanol and stored for 24 h at −20°C. Fixed cells were incubated with RNase A (0.2 mg/ml) (Sigma, St. Louis, MO, USA) at 37°C for 30 min and stained with PI (1 μg/ml) (Sigma, St. Louis, MO, USA). DNA content of cells (20,000 events) was analysed using an ACCURI C6 flow cytometer. Statistical differences between treatments for each phase of the cell cycle were analysed by 2-way ANOVA and Tukey's multiple comparison test. *p* < 0.05 was considered significant.

### Apoptosis assays

The pro-apoptotic activity of [10]-gingerol was analysed by flow cytometry with the PE-Annexin-V Apoptosis Detection Kit (Becton, Dickinson, Franklin Lakes, NJ, USA) or using the TUNEL assay kit (Promega Corporation, USA) according to the manufacturer's instructions and as described in Supplementary methods.

### Caspase expression

Cells were grown to sub-confluence in triplicate wells of a 6-well plate and treated for 6 h with [10]-gingerol (50 μM) or control vehicle (0.1% DMSO). Whole cell lysates were prepared in RIPA buffer (10 mM Tris-HCl, pH 7.4, 150 mM NaCl, 5 mM EDTA, 1% sodium deoxycholate, 1% Triton X-100, 0.1% SDS) and 30 μg proteins loaded onto a 4–12% gradient SDS-PAGE. Protein bands were transferred to a PVDF membrane for 1 h at 100V and the membranes blocked for 1 h in PBS, 0.05% Tween-20, 3% normal goat serum. The following antibodies were used for detection of caspases: caspase-3 (BD Pharmingen, cat# 557035); caspase-8 (NOVUS Biologicals, clone FLICE 4-1-20); caspase-9 (NOVUS Biologicals, clone LAP6 96-2-22). Excess antibodies were washed 3 × 5 min with PBS, 0.05% Tween-20, 0.1% BSA and bound antibodies detected with appropriate horseradish peroxidase-conjugated secondary antibodies. Unbound secondary antibodies were removed by washing as above and specific protein bands developed using ECL reagents (GE Healthcare Life Sciences).

### Metastasis assays

Experimental and spontaneous metastasis assays have been described previously [36, 37]. Depending on the experimental setting, mice were sacrificed on day 12–28 as indicated in the figure legends. Tumour growth was measured thrice weekly and metastatic burden in lung, femur and spine quantitated at endpoint by genomic qPCR using Taqman chemistry (Supplementary methods) [36, 37]. For experiments requiring primary tumour excision, mice were anesthetised by IP administration of ketamine (40 μg/g)/xylazil (16 μg/ml) and primary tumours surgically removed when they reached approximately 0.5 cm^3^ (∼0.5 g) and weighed. Brains were removed, imaged on an IVIS Spectrum and processed for paraffin-embedding and IHC staining (Supplementary methods).

## SUPPLEMENTARY MATERIALS FIGURES



## Abbreviations

7-AAD: 7-Aminoactinomycin D: Analysis of variance
DMEM: Dulbecco's Modified Eagle's Medium
DMSO: Dimethyl Sulfoxide
EDTA: Ethylenediamine Tetraacetic Acid
GFAP: Glial Fibrillary Acidic Protein
H&E: Hematoxylin and Eosin
HER-2: Human Epidermal Growth Factor 2
HPLC: High-Performance Liquid Chromatography
IHC: Immunohistochemistry
IP: Intraperitoneal
MAPK: Mitogen-Activated Protein Kinase
PE-annexin-V: Phycoerythrin-Annexin-V
PI: Propidium Iodide
PVDF: Polyvinylidene Fluoride
qPCR: Quantitative Polymerase Chain Reaction
RIPA: Radio-Immunoprecipitation Assay
SD: Standard Deviation
SRB: Sulforhodamine B
TCA: Trichloroacetic acid
TNBC: Triple negative breast cancer
TUNEL: Terminal Deoxynucleotidyl Transferase dUTP Nick End Labelling.

## References

[1] Stuckey A (2011). Breast cancer: epidemiology and risk factors. Clin Obstet Gynecol.

[2] Pal SK, Childs BH, Pegram M (2011). Triple negative breast cancer: unmet medical needs. Breast Cancer Res Treat.

[3] Dawood S, Broglio K, Esteva FJ, Yang W, Kau SW, Islam R, Albarracin C, Yu TK, Green M, Hortobagyi GN, Gonzalez-Angulo AM (2009). Survival among women with triple receptor-negative breast cancer and brain metastases. Ann Oncol.

[4] Heitz F, Rochon J, Harter P, Lueck HJ, Fisseler-Eckhoff A, Barinoff J, Traut A, Lorenz-Salehi F, du Bois A (2011). Cerebral metastases in metastatic breast cancer: disease-specific risk factors and survival. Ann Oncol.

[5] Allison KH (2012). Molecular pathology of breast cancer: what a pathologist needs to know. Am J Clin Pathol.

[6] Yagata H, Kajiura Y, Yamauchi H (2011). Current strategy for triple-negative breast cancer: appropriate combination of surgery, radiation, and chemotherapy. Breast Cancer.

[7] Deeken JF, Loscher W (2007). The blood-brain barrier and cancer: transporters, treatment, and Trojan horses. Clin Cancer Res.

[8] Steeg PS, Camphausen KA, Smith QR (2011). Brain metastases as preventive and therapeutic targets. Nat Rev Cancer.

[9] Sud S, Lai P, Zhang T, Clemons M, Wheatley-Price P (2015). Chemotherapy in the oldest old: The feasibility of delivering cytotoxic therapy to patients 80 years old and older. J Geriatr Oncol.

[10] Zagar TM, Cardinale DM, Marks LB (2016). Breast cancer therapy-associated cardiovascular disease. Nat Rev Clin Oncol.

[11] Brami C, Bao T, Deng G (2016). Natural products and complementary therapies for chemotherapy-induced peripheral neuropathy: A systematic review. Crit Rev Oncol Hematol.

[12] Radhakrishnan EK, Bava SV, Narayanan SS, Nath LR, Thulasidasan AK, Soniya EV, Anto RJ (2014). [6]-Gingerol induces caspase-dependent apoptosis and prevents PMA-induced proliferation in colon cancer cells by inhibiting MAPK/AP-1 signaling. PLoS One.

[13] Sheu MT, Jhan HJ, Hsieh CM, Wang CJ, Ho HO (2015). Efficacy of antioxidants as a Complementary and Alternative Medicine (CAM) in combination with the chemotherapeutic agent doxorubicin. Integr Cancer Ther.

[14] Semwal RB, Semwal DK, Combrinck S, Viljoen AM (2015). Gingerols and shogaols: Important nutraceutical principles from ginger. Phytochemistry.

[15] Poltronieri J, Becceneri AB, Fuzer AM, Filho JC, Martin AC, Vieira PC, Pouliot N, Cominetti MR (2014). [6]-gingerol as a cancer chemopreventive agent: a review of its activity on different steps of the metastatic process. Mini Rev Med Chem.

[16] Chen CY, Li YW, Kuo SY (2009). Effect of [10]-gingerol on [ca2+]i and cell death in human colorectal cancer cells. Molecules.

[17] Ryu MJ, Chung HS (2015). [10]-Gingerol induces mitochondrial apoptosis through activation of MAPK pathway in HCT116 human colon cancer cells. In Vitro Cell Dev Biol Anim.

[18] Joo JH, Hong SS, Cho YR, Seo DW (2016). 10-Gingerol inhibits proliferation and invasion of MDA-MB-231 breast cancer cells through suppression of Akt and p38MAPK activity. Oncol Rep.

[19] Almada da Silva J, Becceneri AB, Sanches Mutti H, Moreno Martin AC, Fernandes da Silva MF, Fernandes JB, Vieira PC, Cominetti MR (2012). Purification and differential biological effects of ginger-derived substances on normal and tumor cell lines. J Chromatogr B Analyt Technol Biomed Life Sci.

[20] Fuzer AM, Lee SY, Mott JD, Cominetti MR (2017). [10]-Gingerol Reverts Malignant Phenotype of Breast Cancer Cells in 3D Culture. J Cell Biochem.

[21] Ho SC, Chang KS, Lin CC (2013). Anti-neuroinflammatory capacity of fresh ginger is attributed mainly to 10-gingerol. Food Chem.

[22] Fitzgerald DP, Palmieri D, Hua E, Hargrave E, Herring JM, Qian Y, Vega-Valle E, Weil RJ, Stark AM, Vortmeyer AO, Steeg PS (2008). Reactive glia are recruited by highly proliferative brain metastases of breast cancer and promote tumor cell colonization. Clin Exp Metastasis.

[23] Zhang F, Thakur K, Hu F, Zhang JG, Wei ZJ (2017). 10-Gingerol, a Phytochemical Derivative from “Tongling White Ginger”, Inhibits Cervical Cancer: Insights into the Molecular Mechanism and Inhibitory Targets. J Agric Food Chem.

[24] Bernard MM, McConnery JR, Hoskin DW (2017). [10]-Gingerol, a major phenolic constituent of ginger root, induces cell cycle arrest and apoptosis in triple-negative breast cancer cells. Exp Mol Pathol.

[25] Danwilai K, Konmun J, Sripanidkulchai B, Subongkot S (2017). Antioxidant activity of ginger extract as a daily supplement in cancer patients receiving adjuvant chemotherapy: a pilot study. Cancer Manag Res.

[26] Zick SM, Ruffin MT, Lee J, Normolle DP, Siden R, Alrawi S, Brenner DE (2009). Phase II trial of encapsulated ginger as a treatment for chemotherapy-induced nausea and vomiting. Support Care Cancer.

[27] Mukkavilli R, Yang C, Singh Tanwar R, Ghareeb A, Luthra L, Aneja R (2017). Absorption, Metabolic Stability, and Pharmacokinetics of Ginger Phytochemicals. Molecules.

[28] Hamilton A, Sibson NR (2013). Role of the systemic immune system in brain metastasis. Mol Cell Neurosci.

[29] Singh M, Arseneault M, Sanderson T, Murthy V, Ramassamy C (2008). Challenges for research on polyphenols from foods in Alzheimer's disease: bioavailability, metabolism, and cellular and molecular mechanisms. J Agric Food Chem.

[30] Zeng GF, Zhang ZY, Lu L, Xiao DQ, Zong SH, He JM (2013). Protective effects of ginger root extract on Alzheimer disease-induced behavioral dysfunction in rats. Rejuvenation Res.

[31] Zick SM, Djuric Z, Ruffin MT, Litzinger AJ, Normolle DP, Alrawi S, Feng MR, Brenner DE (2008). Pharmacokinetics of 6-gingerol, 8-gingerol, 10-gingerol, and 6-shogaol and conjugate metabolites in healthy human subjects. Cancer Epidemiol Biomarkers Prev.

[32] Denoyer D, Potdevin T, Roselt P, Neels OC, Kirby L, Greguric I, Katsifis A, Dorow DS, Hicks RJ (2011). Improved detection of regional melanoma metastasis using 18F-6-fluoro-N-[2-(diethylamino)ethyl] pyridine-3-carboxamide, a melanin-specific PET probe, by perilesional administration. J Nucl Med.

[33] Kim S, Redvers R, Denoyer D, Pouliot N (2017). Development and validation of a clinically relevant mouse model of breast cancer brain metastasis. 16th Biennial Congress of the Metastasis Research.

[34] Kusuma N, Denoyer D, Eble JA, Redvers RP, Parker BS, Pelzer R, Anderson RL, Pouliot N (2012). Integrin-dependent response to laminin-511 regulates breast tumor cell invasion and metastasis. Int J Cancer.

[35] Sloan EK, Pouliot N, Stanley KL, Chia J, Moseley JM, Hards DK, Anderson RL (2006). Tumor-specific expression of alphavbeta3 integrin promotes spontaneous metastasis of breast cancer to bone. Breast Cancer Res.

[36] Denoyer D, Kusuma N, Burrows A, Ling X, Jupp L, Anderson RL, Pouliot N (2014). Bone-derived soluble factors and laminin-511 cooperate to promote migration, invasion and survival of bone-metastatic breast tumor cells. Growth Factors.

[37] Carter RZ, Micocci KC, Natoli A, Redvers RP, Paquet-Fifield S, Martin AC, Denoyer D, Ling X, Kim SH, Tomasin R, Selistre-de-Araujo H, Anderson RL, Pouliot N (2015). Tumour but not stromal expression of beta3 integrin is essential, and is required early, for spontaneous dissemination of bone-metastatic breast cancer. J Pathol.

