# Supplementary material for: [10]-gingerol induces apoptosis and inhibits metastatic dissemination of triple negative breast cancer in vivo
Source: Oncotarget. 2017 Aug 10;8(42):72260–71. doi: 10.18632/oncotarget.20139 (PMC5641128; doi:10.18632/oncotarget.20139)
Supplement: Supplementary file 1 [file oncotarget-08-72260-s001.pdf]

[10]-gingerol induces apoptosis and inhibits metastatic dissemination of triple negative breast cancer *in vivo*

SUPPLEMENTARY MATERIALS

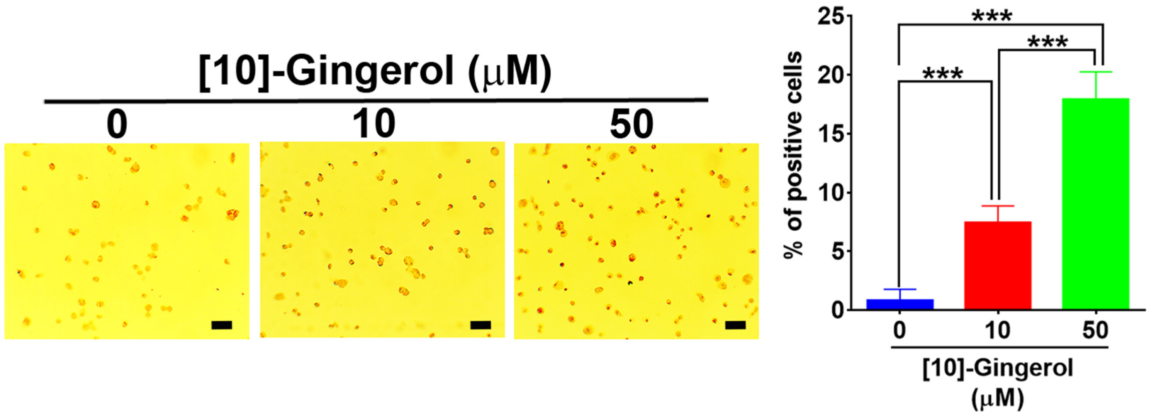

Supplementary Figure 1: [10]-gingerol induces apoptosis in human brain-metastatic MDA-MB-231Br TNBC cells. TUNEL in MDA-MB-231Br. TUNEL-positivity in MDA-MB-231Br cells treated with 0, 10 or 50 µM [10]-gingerol was determined after 18 h treatment as indicated and as described in Supplementary methods. Representative images are shown on the left. Scale bars = 100 µm. The number of positive cells were counted and the data expressed as mean % of positive cells ± SD from six 10× images/condition (right panel). Statistical significance was determined using a 1-way ANOVA, Bonferroni post-test, ***$p < 0.001$.

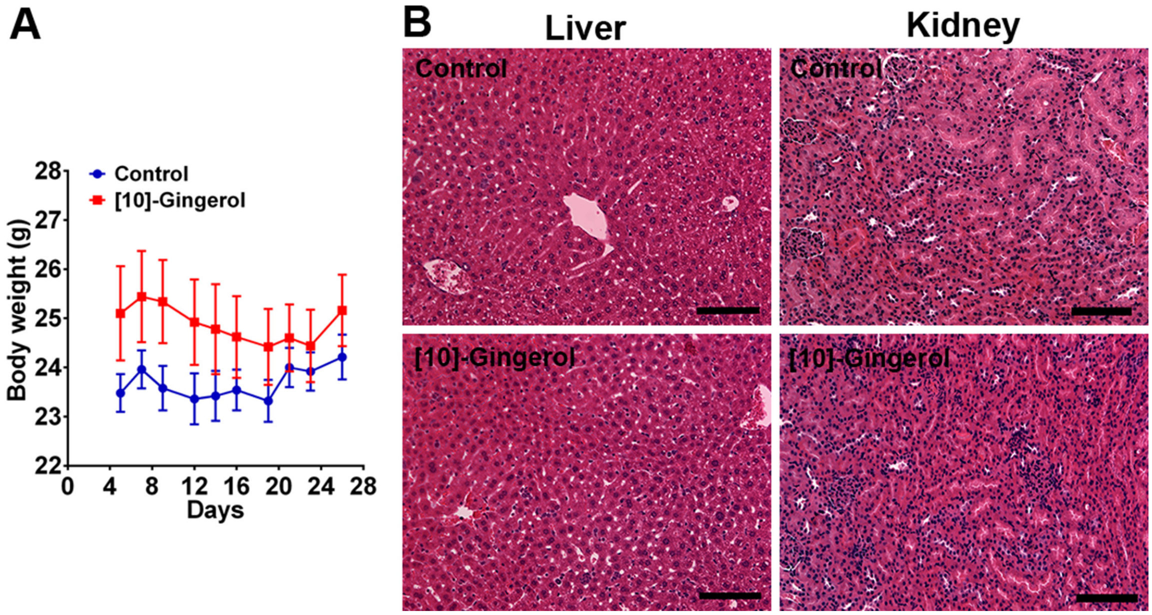

Supplementary Figure 2: [10]-gingerol is well tolerated and non-toxic in 4T1Br4-bearing Balb/c mice. (**A**) Body weight was measured from day 5 to 26. [10]-gingerol (5 mg/kg) was administered daily by intraperitoneal injection from day 9 to day 23. Control and [10]-gingerol-treated mice ($n = 10$/group) showed no significant change in body weight (> 10 %) for the duration of the experiment. (**B**) Histological assessment of liver and kidneys gross morphology in control and [10]-gingerol treated mice. No noticeable morpholological differences were noted between controls and [10]-gingerol-treated mice. Representative images are shown. Scale bar = 100 µm.

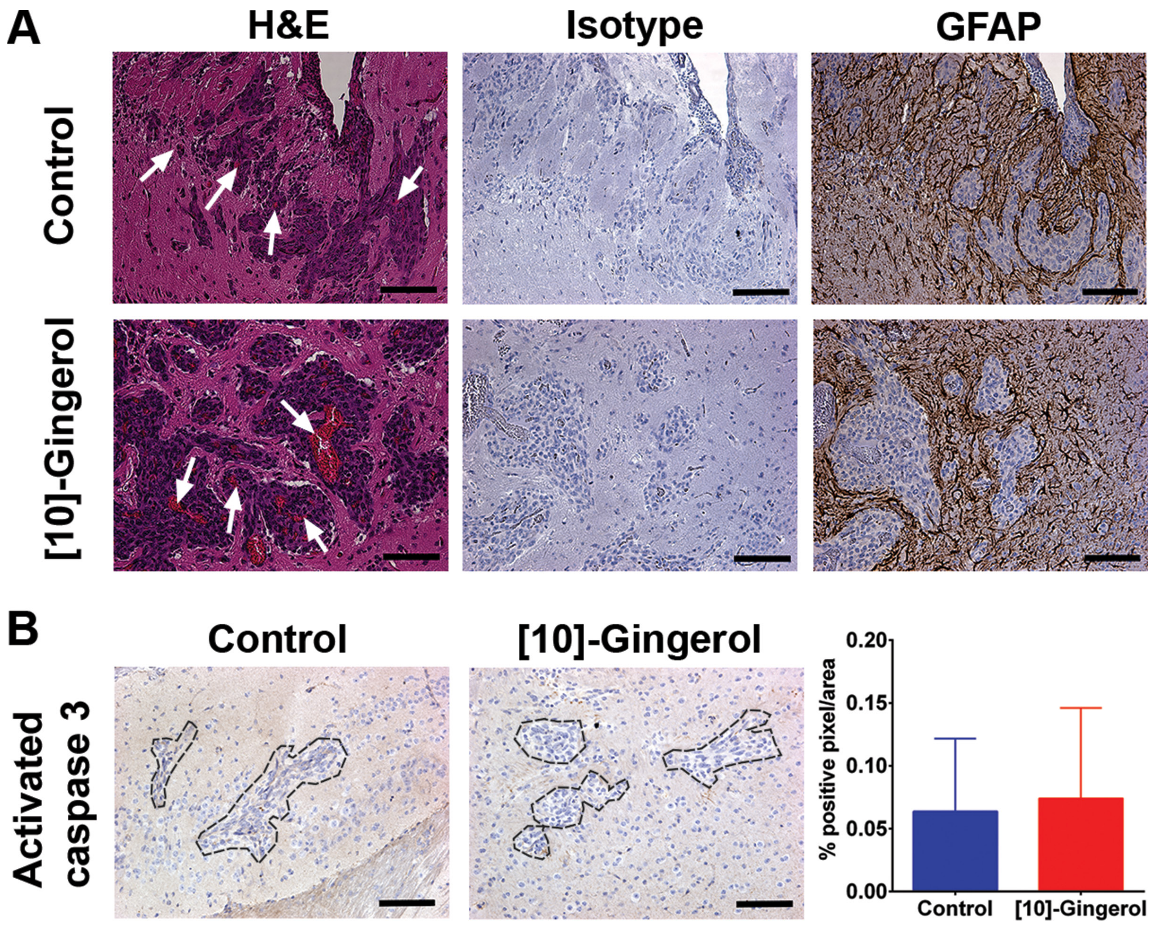

Supplementary Figure 3: [10]-gingerol does not prevent recruitment of a reactive glia or induce caspase-3 activation in established brain lesions. (**A**) Histological staining of brain sections. Representative brain sections with brain metastases from control or [10]-gingerol-treated mice were stained by standard H&E (left) or for IHC detection of GFAP (right) as described in Supplementary methods. Isotype-matched controls are shown in middle panels. Arrows show the presence of blood vessels within or in close proximity of metastatic nodules. Note the strong GFAP reactivity in both control and [10]-gingerol groups. Scale bars = 100 µm. (**B**) IHC staining of activated caspase-3 was carried out as described in Supplementary methods. Note the absence of caspase-3 positivity in control and [10]-gingerol sections. Metastatic nodules are delineated with a dotted line. Scale bar = 100 µm. Caspase-3 quantitation (right panel) from 3 representative brains/group (3 images/section 100µm apart x 3 brains/group = 9 images) was performed using Metamorph quantitation software. Students *t* test, $p = 0.619$.

SUPPLEMENTARY MATERIALS AND METHODS

PE-Annexin-V staining

Cells (1×10^5) were seeded in 12 well plates in complete medium and incubated for 24 h. The medium was removed and the cells incubated in the absence or presence of [10]-gingerol for 8 h at 37°C and 5% CO_2. Cells were harvested by trypsinisation, washed twice with cold PBS and resuspended in binding buffer (100 µl). Cells were incubated with PE-Annexin-V (5 µl) and 7-aminoactinomycin D (7-AAD) (5 µl) for 15 min at room temperature, in the dark. Four hundred µl of binding buffer were added and the cells analyzed on a BD Accuri C6 flow cytometer (Becton, Dickinson and Company, Franklin Lakes, NJ, USA). Specific fluorescence was quantified using CellQuest software (Becton, Dickinson).

TUNEL assay

Briefly, 1×10^6 cells were seeded in 6cm Petri dishes (Corning, USA) and treated with 10 or 50 µM of [10]-gingerol for 18 h. Cells were harvested, washed and resuspended in PBS and seeded onto poly-L-lysine-coated slides. After air-drying for 15 min, the cells were fixed with 4% paraformaldehyde for 25 min at room temperature, washed with PBS, permeabilised with 0.2% Triton X-100 in phosphate-buffered saline (pH 7.4) for 5 min, washed again with PBS and equilibrated with equilibration buffer for 5 min. The end-labelling reaction was performed using compound solution containing biotinylated nucleotide mix, equilibration buffer and terminal deoxynucleotidyl transferase for 1 h at 37°C and terminated with addition of 2 × SSC solution. Cells were washed with PBS and treated with a hydrogen peroxide solution to block endogenous peroxidases. Staining was carried using a streptavidin-HRP solution and diaminobenzidine (DAB) substrate. Slides were mounted in DPX, and visualised under a light microscope (Coleman, N-120).

Tumour growth and metastasis assays

Female Balb/c mice (6–8 week old) were purchased from The Walter & Eliza Hall Institute (Melbourne, Australia) and maintained in a specific pathogen-free environment and fed *ad libitum*. All procedures involving mice conformed to National Health and Medical Research Council animal ethics guidelines and were approved by the Peter MacCallum and State University of Campinas Animal Ethics & Experimentation Committees (Ethics E507 and 3224-1, respectively).

For experimental metastasis assays, mice were anesthetised by isoflurane inhalation and the cells (5×10^4/100 µl PBS) inoculated into the left ventricle of the heart as described [31]. Mice were sacrificed 12 days after tumour cell inoculation and bones (femur and spine) processed for metastatic burden quantitation by genomic qPCR. For spontaneous metastasis assays, cells (1×10^5) were inoculated in the 4th mammary fat pad, tumour growth was monitored thrice weekly with electronic calipers. Lung, femur and spine were harvested at endpoint, snap frozen, homogenised and metastatic burden quantitated by qPCR using Taqman chemistry (PE Biosystems, Foster City, CA, USA). Briefly, a multiplex reaction was performed on a HT7900 cycler to determine the ratio of signal threshold (ΔC_T) between a marker gene (mCherry) present in tumour cells only and vimentin gene present in all cells and the ΔC_T used to calculate the relative tumour burden (RTB) as RTB = $10{,}000/2^{\Delta CT}$. Mouse primers and probes were as follows: vimentin; forward primer 5′- AGC TGCTAACTACCAGGACACTATTG-3′, reverse primer 5′- CGAAGGTGACGAGCCATCTC-3′, probe VIC-CCTT CATGTTTTGGATCTCATCCTGCAGG-TAMRA. mCherry: forward primer 5′- GACCACCTACAAGGCCA AGAAG-3′, reverse primer 5′- AGGTGATGTCCAACTT GATGTTGA-3′, probe 6FAM-CAGCTGCCCGGCGCC TACA-TAMRA.

Histology and IHC staining

Where indicated, primary tumours or metastases were formalin-fixed and paraffin embedded (FFPE), sectioned (4 µm) and processed for standard hematoxylin & eosin (H&E) or IHC staining of caspase-3, Ki67 and glial fibrillar acidic protein (GFAP). For active caspase-3 staining, tissues were subjected to heat antigen retrieval in Tris-EDTA pH 9.0 in a pressure cooker (3 min at 95°C, 10 sec at 90°C). Endogenous peroxidases were inactivated in a solution of 3% H_2O_2 in PBS for 10 min and the slides washed twice in water and once in PBS before blocking for 30 min in PBS, 2% BSA. Sections were reacted overnight at 4°C with a rabbit anti-active caspase-3 antibody (Cell Signalling Technology #9664, 1/100 dilution in PBS, 2% BSA) or rabbit anti-Ki67 (Abcam, Ab15580). Sections were washed 3 × 5 min in PBS to remove unbound antibodies and biotin-conjugated goat anti-rabbit secondary antibodies (DAKO # E0432, 1/200 dilution if PBS, 2% BSA) added for 30 min at room temperature. The slides were washed as above, incubated with ABC reagents (Vectastain kit #PK6100) for 30 min at room temperature and washed as above. Specific binding was detected using a DAB peroxidase substrate kit (Vector Laboratories).

For GFAP staining, antigen retrieval was performed in a microwave for 10 min in citrate buffer, pH 6.0. Sections were washed briefly with PBS followed by washing with PBS, 0.3% Triton X-100 for 10 min and washing 2 × 3 min with PBS. Sections were then blocked in PBS, 3% normal horse serum for 45 min at room temperature before addition of primary rabbit anti-GFAP antibodies (DAKO #Z0334, 1/500 dilution) overnight at 4°C. Slides were washed 2 × 2 min with PBS, secondary biotin-conjugated goat anti-rabbit secondary antibodies

(DAKO # E0432, 1/200 dilution if PBS, 2% BSA) were added for 60 min at room temperature and excess antibodies removed by washing twice with PBS. Specific binding was detected using ABC reagents and DAB substrate kit as described above.

All sections were counterstained with hematoxylin, dehydrated and mounted in DPX. Sections were photographed using an Olympus BX-51 light microscope and, where indicated, the extent of staining quantitated using Metamorph software. Data are expressed as mean% of positive pixels/field of view ± SD from a total of 27 images/experimental group (3 images/section × 3 section/tumour 150 µm apart × 3 tumours/group). The statistical difference between groups was analyzed using the Student's *t* test; $p < 0.05$ was considered significant.
